# How do the hierarchical levels of premises affect category-based induction: diverging effects from the P300 and N400

**DOI:** 10.1038/s41598-017-11560-y

**Published:** 2017-09-18

**Authors:** Yi Lei, Xiuling Liang, Chongde Lin

**Affiliations:** 1 College of Psychology and Sociology, Shenzhen University, Shenzhen, 518060 China; 2 Shenzhen Key Laboratory of Affective and Social Cognitive Science, Shenzhen, 518060 China; 3 Center for Language and Brain, Shenzhen Institute of Neuroscience, Shenzhen, 518057 China; 4 School of Medicine, Shenzhen University, Shenzhen, 518060 China; 5 School of Psychology, Beijing Normal University, Beijing, 100875 China

## Abstract

Although a number of studies have explored the time course of category-based induction, little is known about how the hierarchical levels (superordinate, basic, subordinate) of premises affect category-based induction. The EEG data were recorded when nineteen healthy human participants were performing a simplified category-based induction task. The ERP results showed that: in the subordinate conclusion condition, the basic premise elicited a larger N400, versus the superordinate promise; in the basic conclusion condition, the superordinate promise elicited a larger P300 relative to both the basic premise and subordinate premise; in the superordinate conclusion condition, however, no difference was found between different promise. Furthermore, the process that reasoning from a higher level to a lower level evoked a larger P300, compared to it did in the reverse direction. The divergent evidence suggested that category-based induction at superordinate, basic, and subordinate levels might be affected by various factors, such as abstract level, direction, and distance between premise and conclusion, which yielded new insights into the neural underpinnings of category-based induction with different inductive strengths.

## Introduction

The ability to extract category knowledge of human brain when they encounter with concepts^1–3^ is foundamental to category-based reasoning. When you see a picture of a dog, you may activate the category knowledge of mammal (e.g., breathe with its lungs, breast-feeding, viviparous), and categorise it as a mammal. Based on conceptual processing and knowledge about these categorise or concepts, the cognitive process helps you reason and come to a certain conclusion. In fact, this process has been investigated within a category-based reasoning task^4,5^, in which a premise item with one property is presented before a conclusion item, and participants are required to judge whether, or not, the conclusion item and the premise item have the same property.

According to previous studies, there are three different levels of abstraction of category, namely “subordinate level”, “basic level”, and “superordinate level”, such as robin-bird-animal^6^. The greater the inclusiveness of a category within a taxonomy, the higher the level of abstraction. Extending this finding to category-based reasoning tasks, participants are presented with a premise sentence: ‘All robins have the property X’ and are informed to judge the probability as to whether ‘All animals/birds/sparrows have property X’, or not. On the contrary, participants are presented with a premise sentence ‘All animals have the property X’ and are required to judge the probability whether ‘All birds/robins have property X’. Due to the previous findings^7,8^, the inductive strength and confidence of participants’ responses in the former case are lower than that in the latter. We can conclude that the abstraction levels of categories included in arguments play an important role in category-based reasoning.

A limitation of existing event-related potential (ERP) studies exploring the time course of category-based induction is that they mainly used pictorial stimuli^9–12^, only a few studies have explored this issue via languages of sentential stimuli^4,5,13^. However, a study by Liang *et al*.^13^ employed sentential stimuli where participants were presented with two premises (*e.g*., S1: the liver of a sparrow has some component X; S2: the liver of a glede has some component X) and one conclusion (Congruent induction: the liver of all birds has some component X; Incongruent induction: the house cat has 32 teeth). They found that congruent induction elicited a larger frontal N400 and a significant increase in the power of the gamma-band compared to incongruent induction, which reflected the dynamics of semantic information integration. Furthermore, Long *et al*.^4^ extended this work and revealed that unrelated-category conclusion condition (*e.g*., the apples have component × 1 → the pens have component × 1), evoked smaller P3b and larger N400 than the related- category conclusion condition (*e.g*., the apples have component × 1 → the pears have component × 1). More recently, Liang *et al*.^5^ found that a larger P3 effect was elicited in typical conclusions (*e.g*., the bird has property X → the sparrows have property X) relative to atypical conclusions (*e.g*., the bird has property X → the penguins have property X) in general premise conditions, which reflected the attentional resource allocation needed for reasoning^14^.

Performing a reasoning task via sentential stimuli implicates both semantic^15,16^ and syntactic processes^17,18^, however, which possibly interfere with the process of category-based induction. Thus, Lei *et al*.^19^ developed a simplified category-based deduction task (*e.g*., Premise: Birds have property X; Conclusion: Sparrows), in which the conclusion was represented by a category member (*e.g*., Sparrows, which means that sparrows have property X). Following this, Long *et al*.^4^ developed a simplified category-based induction task (*e.g*., Premise: Birds × 1; Conclusion: Sparrows × 1?), in which the premise was represented by a category member and a blank property (*e.g*., Apple × 1, which means that apple has property × 1), while the additional “?” was shown at the end of the conclusion (*e.g*., banana × 1?)^5^. Taking these into consideration, we further simplified the task by dissociating the property from the premise and conclusion. In the present study, the premise and conclusion were represented by category members while the property was presented separately between them (*e.g*., Premise: Birds; Property: X; Conclusion: Robins). This way of presenting reasoning tasks had certain advantages, such as reducing the impact of knowledge and sentence processing. Although a number of studies have explored the time course of category-based induction^4,5,13^, little is known about how the hierarchical levels of premises affect category-based induction. In the current study, we set out to address this question by recording and analyzing ERPs.

Evidence from several previous studies implicated that N400 were sensitive to category-based induction. Precisely, the N400 is a negative wave peaking at about 400 ms post-stimulus presentation and likely to be observed in the frontal region. The amplitudes of the N400 is modulated by contextual factors during an anomalous sentence task^15^ or a category verification task^6^. Further studies demonstrated that N400 is related to lexical processing^20^ or semantic processing^21–23^. It has been established in several paradigms that atypical words elicited a larger N400 than typical words during a category verification task^24^, and an auditory category member verification task^25^.

In addition, previous studies report that the P300 is a positive wave peaking at about 350–500 ms post-stimulus presentation which can be recorded in the centro-parietal region^26^: the amplitudes thereof may reflect categorisation processing^27–29^ or be associated with the information-processing cascade related to attentional and memory mechanisms^26,30^. For example, the latency and peak of P300 elicited by typical items were shorter than atypical ones during a category verification task^31^. Moreover, recent reasoning studies proved that matched argument evoked a larger P300 than mismatched ones, indicating the satisfaction of expectations^4,32–35^. A larger P300 had also been found for related-category conclusion condition than for unrelated-category conclusion condition^4^.

Based on the aforementioned considerations, the main purpose of this study was to explore how hierarchical levels of premises modulated category-based induction at behavioural and electrophysiological levels. To achieve this, three hierarchical levels of premises and conclusions (superordinate, basic, and subordinate) were manipulated: in the subordinate conclusion condition, participants are asked to reason separately from three different level categories premises (superordinate, basic, and subordinate) to the same subordinate level category conclusion (*e.g*., Animal/Bird/Sparrow → Robin). In the basic conclusion condition, participants are asked to reason separately from three different level categories premises (superordinate, basic, and subordinate) to the same basic level category conclusion (*e.g*., Animal/Bird/Sparrow → Insect). In the superordinate conclusion condition, participants are asked to reason separately from two different level categories premises (basic and subordinate) to the same basic level category conclusion (*e.g*., Bird/Sparrow → Animal). Participants are required to assume that the information about the premise is true (*e.g*., Bird has property X), and to assess whether, or not, the information about the conclusion (*e.g*., Sparrow has property X) is plausible, and make a “strong” or “not strong” response, which has been used extensively elsewhere^5,7,36^.

We mainly focus on the electrophysiological data elicited by the conclusion(s), where certain judgments could be made. According to the postulate of the model^37^, connections between concepts are different, which is influenced by both the lengths and strength of the nodes. Prior studies find that concepts with hierarchical levels, superordinate, basic, and subordinate concepts play different roles in categorisation^38,39^. Hence, during the performance of reasoning, short RTs and/or stronger plausibility judgments should be found when the connection between two concepts included in the premise and conclusion was stronger. Based on previous studies, furthermore, the process that reasoning from a higher to a lower level should produce stronger plausibility judgments than it does in the reverse direction^7,36^.

Several related ERP experiment have revealed that hierarchical levels of categories have a pervasive influence on categorisation^39,40^. In view of previous reports^4,5,13,19^, we here predict the level of abstraction of premises might affect category-based induction by measuring the N400 amplitude. As for the hierarchical direction during the category-based induction task, another prediction is that it could affect inductive reasoning, which can be reflected in the modulation of P300 amplitudes. Specifically, based on previous studies, a larger frontal N400^13^, as well as a larger P300 on centro-parietal region^4,5^, should be found for the condition with stronger plausibility judgments.

## Method

### Participants

Nineteen healthy, right-handed volunteers (eleven females), between the age of 19 and 26 (21.58 ± 1.96, mean ± SD), took part in the main experiment. All volunteers reported normal, or corrected-to-normal, vision and normal colour perception. All volunteers provided written informed consent and were paid for their participation. In addition, all volunteers were unaware of the experimental purpose of the experiment. All experimental protocols were approved by the University’s ethics committee (The Medicine Medical Ethics Committee of Shenzhen University), and the methods complied with the relevant guidelines and regulations.

### Ethics Statement

The study was approved by the University’s ethics committee (The Medicine Medical Ethics Committee of Shenzhen University).

## Materials

The normed materials were adopted from our previous study^19^. In short, the experiment materials are object names belonging to three levels of abstraction in two taxonomies (plant and animal). The familiarity and typicality^41^ of the materials were evaluated. Moreover, the mean word frequency and word lengths were controlled based on a current Chinese language database (Centre for Chinese Linguistics PKU, China). The detailed results about the normed materials were described in previous study.

As shown in Table 1, ten subordinate level categories for each basic level category, as well as four basic level categories (bird, insect, vegetable, and fruit) and two superordinate categories (animal and plant), were chosen for use as experimental stimuli. Furthermore, forty members of the inanimate category were chosen for use as control stimuli to avoid the fixed response tendencies.

**Table 1:** Normed materials used in the experiment.

Hierarchy of class concept	Materials
Superordinate level	Animal, plant
Basic level	fruit, vegetable, tree, grass, flower, bird, insect, cat, dog, pig, snake, rabbit, fish, tortoise, frog, monkey, cattle, bear, mouse, and tiger
Subordinate level	**Bird**: swallow, magpie, sparrow, oriole, kingfisher, tit, crow, wild geese, pigeon, and lark
	**Insect**: cockroach, ladybug, cricket, grasshopper, beetle, butterfly, bee, dragonfly, fly, and locust
	**Fruit**: apple, orange, pear, peach, watermelon, banana, pineapple, tangerine, grape, and strawberry
	**Vegetable**: cabbage, green vegetable, spinach, radish, cauliflower, eggplant, cucumber, lettuce, pepper, and leeks
	**Non-life**: chair, sofa, bookcase, stool, bureau, tea table, dining table, mattress, desk, kettle, air-condition, fan, loudspeaker, computer, camera, refrigerator, eraser, schoolbag, pencil, pen, glue, chalk, scissor, blackboard, knife, ink, ruler, cap, shoe, trousers, socks, shirt, skirt, scarf, sweater, car, train, steamship, plane, and passenger car

Note The non-life categorisation is used as a control condition.

### Experimental design and task

The present experiment used a single premise category-based induction task with a blank property which is represented by capital letters ranging from A to Z, in order to reduce the memory load and background knowledge effect^2,4,5^. The premise and conclusion both consisted of one of above selected categories. As mentioned earlier, the hierarchical levels of premises and conclusions (superordinate, basic, and subordinate) were manipulated. Considering that only two types of superordinate categorisations were included, the reasoning condition from superordinate to superordinate levels was eliminated. Furthermore, considering that all subordinate categorisations belonged to categorisation of living things, a control condition was added to avoid any tendency to a fixed response. For the control condition, specifically, an inanimate categorisation was used as the conclusion item but the premise item respectively belonged to the subordinate, basic, and superordinate level categorisations from above.

As shown in Table 2, the subordinate conclusion condition included three types of argument: (1) Superordinate-Subordinate (Sup-Sub), the premise consisted of a superordinate level category and the conclusion consisted of a subordinate level category. (2) Basic-Subordinate (Bas-Sub), in which the premise consisted of a basic level category and the conclusion consisted of a subordinate level category; (3) Subordinate-Subordinate (Sub-Sub), the premise and conclusion consisted of different subordinate level categories; Furthermore, the basic conclusion condition also included three types of argument: (1) Superordinate-Basic (Sup-Bas), the premise consisted of a superordinate level category and the conclusion consisted of a basic level category. (2) Basic-Basic (Bas-Bas), the premise and conclusion consisted of different basic level categories; (3) Subordinate-Basic (Sub-Bas), in which the premise consisted of a subordinate level category and the conclusion consisted of a basic level category; however, only two types of arguments were included in the superordinate conclusion condition: (1) Basic-Superordinate (Bas-Sup), the premise consisted of a basic level category and the conclusion consisted of a superordinate level category. (2) Subordinate-Superordinate (Sub-Sup), the premise consisted of a subordinate level category and the conclusion consisted of a superordinate level category; however, the control conditions were as follows: (1) Superordinate-Non-living (Sup-Non), the premise consisted of the superordinate level category and the conclusion consisted of a non-living object. (2) Basic- Non-living (Bas-Non), in which the premise consisted of a basic level category and the conclusion consisted of a non-living object; (3) Subordinate- non-living (Sub-Non), the premise consisted of a subordinate level category and the conclusion consisted of a non-living object.

**Table 2:** The main conditions and examples used in the experiment.

Conditions	Arguments	Premise	Conclusion	Examples		
				Premise	Properties	Conclusion
Subordinate conclusion	Sup-Sub	Superordinate	Subordinate	Animal	X	Sparrow
	Bas-Sub	Basic	Subordinate	Bird	X	Sparrow
	Sub-Sub	Subordinate	Subordinate	Crow	X	Sparrow
Basic conclusion	Sup-Bas	Superordinate	Basic	Animal	Y	Bird
	Bas-Bas	Basic	Basic	Insect	Y	Bird
	Sub-Bas	Subordinate	Basic	Crow	Y	Bird
Superordinate conclusion	Bas-Sup	Basic	Superordinate	Bird	Z	Animal
	Sub-Sup	Subordinate	Superordinate	Crow	Z	Animal
Control	Sup-non-life	Superordinate	Non-life	Animal	O	Sofa
	Bas-non-life	Basic	Non-life	Bird	O	Sofa
	Sub-non-life	Subordinate	Non-life	Crow	O	Sofa

The stimuli were presented using E-Prime software (Psychology Software Tools, Inc. Pittsburgh, PA) on a 17-inch (28 cm) computer monitor against a grey screen. All words were presented in Chinese (Song Ti font, size 64). The viewing distance was approximately 60 cm. Responses were recorded by using a standard QWERTY keyboard. The procedure was divided into practice and test phases. In the practice phase, participants completed a training block with 40 trials to get accustomed to the task condition. The tests phases consisted of 640 trails for main experimental conditions (80 trials for each condition) and 80 trails for the control condition: there were 720 experimental trials (presented randomly) in total, which were divided into six blocks with 120 trials per block. However, the data recorded in the control condition were excluded from analyses. In this case, ERP responses to conclusion items onsets and behavioural responses of eight experiment conditions were analyzed.

**Figure 1:**
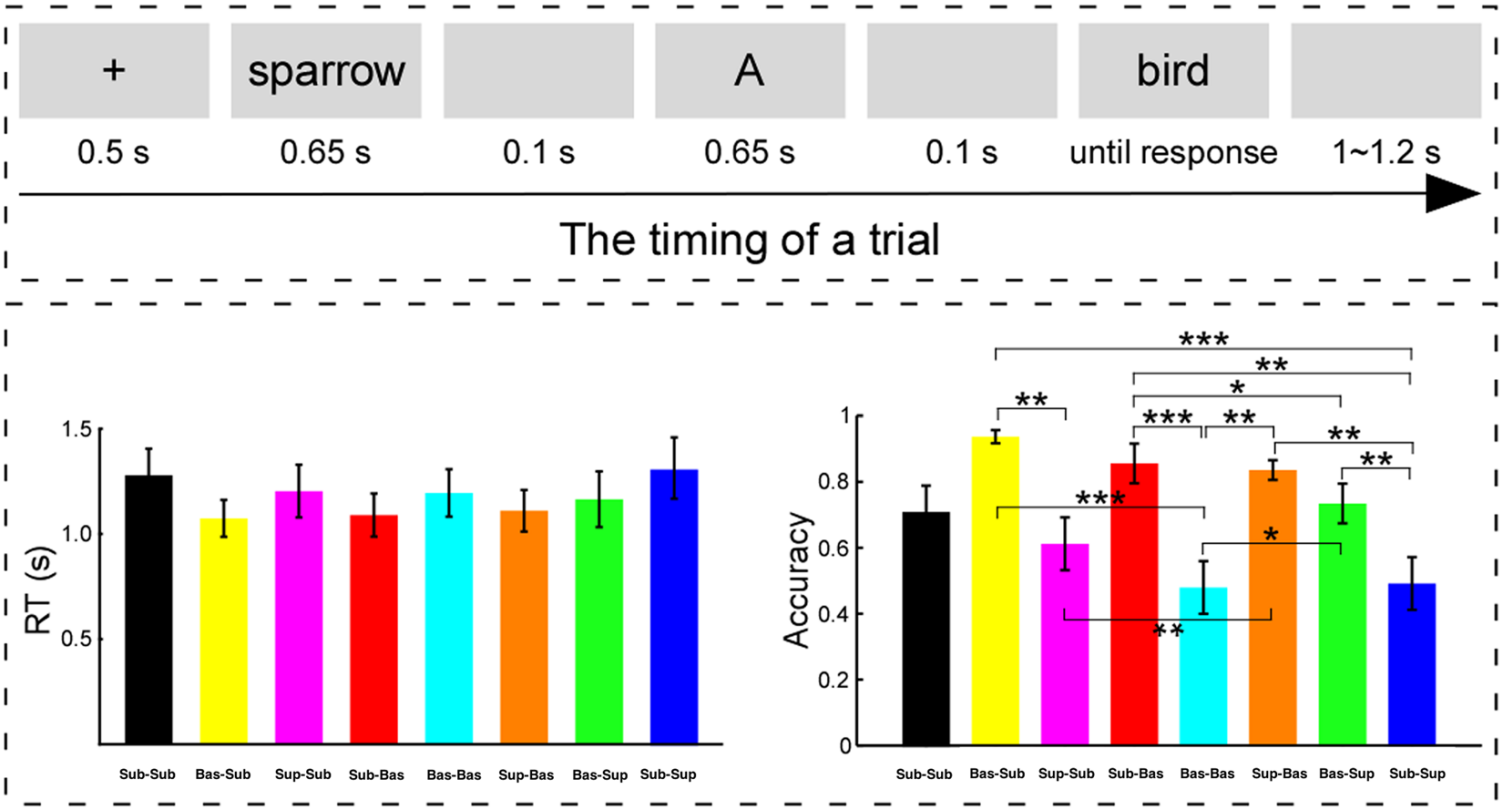
Experimental procedure of the categorisation reasoning task and behavioural performance. **Top**: A representative sequence and the detailed timing of one trial. Note that the illustration depicted a subordinate-basic categorisation. Moreover, ‘sparrow’ was a typical representation of ‘bird’. **Bottom left**: The mean RT in the conclusion items. It was notable that RT results did not reveal significant difference among the subordinate-subordinate, basic-subordinate, superordinate-subordinate, subordinate-basic, basic-basic, superordinate-basic, basic-superordinate, and subordinate-superordinate categorizations, *p* > 0.05 (one-way repeated-measures ANOVA). **Bottom right**: The accuracy of positive judgment in the conclusion items. The results revealed significant difference among the eight categorizations, *p* < 0.001 (one-way repeated-measures ANOVA). Note: In the bottom panel, *p < 0.05, **p < 0.01, and *p* < 0.001, respectively, *N* = 19. RT is response time; For each trial type, error bars represent ± SEM across all participants.

As shown in Fig. 1 (top), in each trial, the stimuli were presented as follows: (1) a fixation for 500 ms; (2) a premise item for 650 ms; (3) a blank interval for 100 ms; (4) a letter ranging from A to Z which represented property of the premise item for 650 ms; (5) a blank interval for 100 ms; (6) a conclusion item lasting until a key was pressed, where participants made their judgement about the probability (strong, not strong) that the conclusion item and premise item had the same property and pressed the ‘F’ or ‘J’ key with their left or right forefinger respectively, or pressed the space bar if failed to decide which one to choose; and (7) a blank interval for 1000 to 1200 ms (the interval was varied at random within this range). The key press (‘F’ or ‘J’) was counterbalanced across participants, and they were instructed to perform the task as fast as possible without sacrificing accuracy. Participants were allowed to rest themselves between blocks and continue the experiment by pressing any key.

### EEG recording and analysis

#### EEG recording

The EEG data were recorded by a 64-channel Brain Products system (Brain Products GmbH, Munich, Germany; pass band: 0.01–100 Hz, sampling rate: 500 Hz) which used a standard EEG cap based on the extended 10–20 system. The left mastoid was used as the reference channel, and all channel impedances were kept below 5 kΩ. The electro-oculographic (EOG) signals were simultaneously recorded from four surface electrodes, which were placed over the upper and lower eyelids and laterally 1 cm from the outer corner of the left and right orbits to monitor ocular movements and eye blinks. All data are available by contacting with us.

#### EEG data pre-processing

The EEG data were pre-processed using EEGLAB^42^, an open source toolbox running under the MATLAB™ environment. The EEG trials were re-referenced to a common average reference. Continuous EEG data were bandpass filtered between 1 and 30 Hz. EEG epochs were segmented in 1200 ms time-windows (pre-stimulus 200 ms and post-stimulus 1000 ms) and baseline corrected using the pre-stimulus time interval. Trials contaminated with EOG artifacts (mean EOG voltage exceeding ± 80 μV) or those with artifacts due to amplifier clipping, bursts of electromyographic (EMG) activity, or a peak-to-peak deflection exceeding ± 80 μV were excluded from analysis. The remaining EOG artifacts were subtracted using a validated method based on independent component analysis (ICA)^42–44^. In all datasets, the independent components (ICs) related to eye movements had a large EOG channel contribution and a frontal scalp distribution. Then, date were visually inspected to identify bad epochs which were rejected from further analysis.

#### ERP analyses

For each participant and each trial type, average waveforms were computed, and time-locked to the onsets of the conclusion items. Single-participant average waveforms were subsequently averaged to obtain group-level average waveforms. For each condition, N400 mean amplitudes of each participant were measured at the centro-frontal [(Fz + F1 + F2 + FCz + FC1 + FC2)/6] region between 290 ms and 410 ms, and P300 mean amplitudes of each participant were measured at the centro-parietal region [(CP1 + CPz + CP2 + P1 + Pz + P2)/6] between 240 ms and 410 ms. The chosen electrodes and time windows matched the strongest N400 and P300 activities of the current data and were similar to those found in previous research^19,23,26,45^. Moreover, averaging across multiple electrodes decreased the chance of spurious findings by increasing the signal-to-noise ratio^46^. The resulting mean amplitudes were respectively compared using (1) the one-way repeated-measures analysis of variance (ANOVA) in the eight conditions and (2) the paired-samples *t* test to investigate the distance effect and direction effect of reasoning, respectively. The group-level scalp topographies in the N400 and P300 time windows for the eight conditions were obtained, respectively.

## Results

### Behavioural performance

The behavioural analyses focused on the mean RT and the proportion of ‘strong’ response. Across participants, the RT and the accuracy of the positive judgment for the eight experimental conditions are shown in Fig. 1 (bottom left).

Firstly, we conducted eight-level one-way repeated-measures ANOVA for the RT. Mauchly’s test was applied to assess the possible violations of sphericity^47^. If the sphericity assumption was violated (*p* < 0.05), the number of degrees of freedom was corrected according to the Greenhouse–Geisser method^48^. The results showed a non-significant difference among the eight levels, *F* (3.36, 60.03) = 2.18, *p* > 0.09, *η* ^2^ = 0.11, indicating there were no remarkable behavioural differences among the eight conditions and, therefore, the RT data were not analysed further.

Secondly, the same analyses were conducted for the proportion of ‘strong’ response. The one-way repeated-measures ANOVA results showed a significant difference among the eight conditions, *F* (2.94, 53.01) = 11.24, *p* < 0.001, *η* ^2^ = 0.38. As shown in Fig. 1 (bottom right), a *post hoc* test found that strongest plausibility judgments were found for ‘basic-subordinate’ and ‘superordinate-basic’, which were significantly larger than ‘superordinate-subordinate’, ‘basic-basic’, and ‘subordinate-superordinate’ conditions, with *p*s < 0.05. Furthermore, the plausibility for ‘subordinate-basic’ was significantly larger than ‘basic-superordinate’ condition, while both of them were stronger than in ‘basic-basic’ and ‘subordinate-superordinate’ conditions, with *p*s < 0.05. Finally, the plausibility for ‘subordinate- subordinate’ was larger than in the ‘basic-basic’ condition, with *p*s < 0.05.

### ERP data

**Figure 2:**
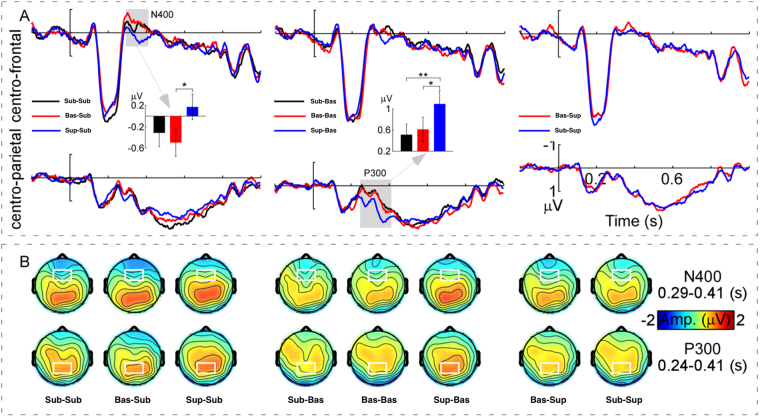
Group-level average ERPs, mean amplitudes, and scalp topographies of N400 and P300 waves. Panel A (Top): The grand-average ERP waveforms measured at the centro-frontal region [(Fz + F1 + F2 + FCz + FC1 + FC2)/6] for the subordinate-subordinate, basic-subordinate, superordinate-subordinate, subordinate-basic, basic-basic, superordinate-basic, basic-superordinate, and subordinate-superordinate categorisations. Note that when the conclusion items were subordinate categorisations, N400 amplitudes were modulated by the categorisations of premise items (subordinate, basic, and superordinate) in the time window from 0.29–0.41 s (outlined by the grey rectangle). Panel A (Bottom): The grand-average ERP waveforms measured at the centro-parietal region [(CP1 + CPz + CP2 + P1 + Pz + P2)/6] for the eight trial types. It is notable that when the conclusion items belonged to basic level categorisations, P300 amplitudes were modulated by the categorisations of premise items (subordinate, basic, and superordinate) in the time window from 0.24–0.41 s (outlined by the grey rectangle). However, when the conclusion items belonged to superordinate categorisations, neither N400 nor P300 amplitudes were modulated by the categorisations of premise item (subordinate and basic). *X*-axis, time (s); *Y*-axis, amplitude (μV). The vertical bars indicate the onsets of conclusion items. The inlayed histograms intuitively show the N400 and P300 amplitudes as indicated by the grey arrows. Error bars indicate ± 1 standard error of the mean (SEMs). Note: *p < 0.05 and **p < 0.01, respectively, *N* = 19. Panel B shows the scalp topographies of N400 (averaged within 0.29–0.41 s) and P300 (averaged within 0.24–0.41 s) for the eight trial types, respectively. Noteworthy was that the scalp topographies of N400 and P300 displayed clear centro-frontal and centro-parietal distributions (marked in white) for all trial types, respectively. Note: ‘Amp’ is amplitude.

Figure 2A shows the grand-average ERP waveforms measured at the centro-frontal [(Fz + F1 + F2 + FCz + FC1 + FC2)/6] and centro-parietal [(CP1 + CPz + CP2 + P1 + Pz + P2)/6] regions and N400 and P300 amplitudes measured from 290 ms to 410 ms and from 240 ms to 410 ms after onsets of conclusion items, respectively. As can be seen in Fig. 2A, the onsets of the conclusion items (1) elicited a dominant N400 between 290–410 ms (highlighted by a grey rectangle) distributed in the centro-frontal region for the subordinate-subordinate, basic-subordinate, and superordinate-subordinate conditions; and (2) elicited a marked P300 between 240–410 ms (highlighted by a grey rectangle) distributed in the centro-parietal region for the subordinate-basic, basic-basic, and superordinate-basic conditions; however, such N400 and P300 effects were insignificant for the basic-superordinate and subordinate-superordinate conditions.

Figure 2B shows the scalp topographies of N400 (top, 290–410 ms) and P300 (bottom, 240–410 ms) for the eight conditions, respectively: as seen in Fig. 2B, the N400 amplitude difference was significant among the subordinate-subordinate, basic-subordinate, and superordinate-subordinate conditions in the centro-frontal regions (marked by white rectangles) and the P300 amplitude difference was significant among the subordinate-basic, basic-basic, and superordinate-basic conditions in the centro-parietal region (marked by white rectangles). However, both the N400 and the P300 amplitudes were no significantly different in basic-superordinate and subordinate-superordinate conditions.

### Distance effect of reasoning

Consistent with the behavioural analyses, we conducted the eight-level one-way repeated-measures ANOVA for the N400 mean amplitudes in subordinate- subordinate, basic-subordinate, superordinate-subordinate, subordinate-basic, basic-basic, superordinate-basic, basic-superordinate, and subordinate-superordinate conditions: the criterion used to correct the number of degrees of freedom was the same as that used for analysis of behavioural data. The results showed a significant difference among the eight levels, *F* (7, 26) = 3.47, *p* < 0.01, *η* ^2^ = 0.16. A *post hoc* test revealed significantly more negative amplitudes for basic-subordinate relative to superordinate-subordinate condition, *p* < 0.05; and did not reveal any other significant difference, *p*s > 0.10 (Bonferroni correction).

Similarly, for the P300 mean amplitudes, the eight-level one-way repeated-measures ANOVA was performed. The results showed a significant difference among the eight levels, *F* (7, 26) = 6.70, *p* < 0.001, *η* ^2^ = 0.27. A *post hoc* test revealed significantly more positive amplitudes for superordinate-basic relative to both basic-basic and subordinate-basic conditions, *p* < 0.05 and *p* < 0.01, respectively. In addition, there were no other significant differences found, with *p*s > 0.10.

### Direction effect of reasoning

To investigate the direction effect of reasoning, for the N400 and P300 mean amplitudes, we performed paired-samples *t* tests (two-tailed) (1) between subordinate-basic and basic-subordinate conditions, (2) between basic-superordinate and superordinate-basic conditions, and (3) between subordinate-superordinate and superordinate-subordinate conditions.

For the N400 mean amplitudes, there was no significant difference found, with *p*s > 0.05.

**Figure 3:**
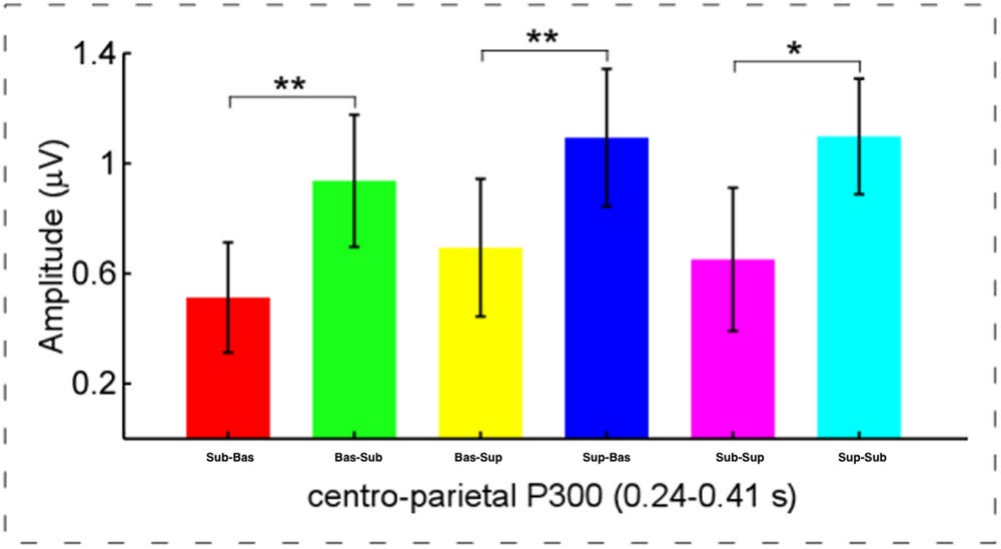
Illustration of P300 amplitudes to reveal the direction effect of categorisations. It was notable that modulation of P300 amplitudes in the centrol-parietal region in the time widow of 0.24–0.41 s after the onsets of the conclusion items revealed the obvious direction effect in categorisations. Note: Error bars indicate ± 1 standard error of the mean (SEMs), *p*p < 0.05, **p < 0.01. *N* = 19.

As shown in Fig. 3, for the P300 mean amplitudes, the results revealed marked differences (1) between subordinate-basic and basic-subordinate conditions, *t*(18) = 3.42, *p* < 0.01, (2) between basic-superordinate and superordinate-basic conditions, *t*(18) = 3.51, *p* < 0.01, and (3) between subordinate-superordinate and superordinate-subordinate conditions, *t*(18) = 2.76, *p* < 0.05. Furthermore, the process that reasoning from a higher level to a lower level evoked a larger P300, compared to it did in the reverse direction (i.e., basic-subordinate condition > subordinate-basic condition; superordinate-basic condition > basic-superordinate condition; superordinate-subordinate condition > subordinate-superordinate condition).

## Discussion

The present study was designed to investigate the behavioural and brain characteristics of category-based induction at different hierarchical levels of abstraction. The results showed that no significant difference was found for the RT in the eight conditions. The non-significant effect of condition on RTs might be due to the “non-strong” response to some conditions or the “pressing the space key” in some conditions. However, we may partially illustrate that due to the nonsignificant difference on the RTs, the experiment conditions used in this study were regarded as equally difficult. However, significant differences in plausibility were found for the proportions of plausibility judgment among conditions, which embodied the differences in the level of generalisation of the premises in inductive reasoning. Generally speaking, the greater distance between categorisations usually led to a lower plausibility in category-based induction.

The first finding is that when the promise item and conclusion item are at the same hierarchical level, the plausibility in the basic-basic condition was significantly lower than that in the subordinate-subordinate condition. According to the spreading activation model in semantic networks^49^, along with all of the subordinate categories are the most typical member of four basic categories, the connections between subordinate and subordinate nodes were stronger than those between basic and basic nodes and the distance between different basic categories belonging to the same superordinate category are greater than that between different subordinate categories belonging to the same basic category. This view was further supported by the low plausibility in basic-basic conditions relative to other arguments composed by basic and subordinate items, or basic and superordinate items with different directions, such as basic-subordinate, subordinate-basic, basic-superordinate, and superordinate-basic conditions. In fact, these result can also be explained from the view of the two forms of inductive reasoning: specific conclusions (*e.g*., sparrow-robin) and general conclusions (*e.g*., sparrow-bird)^50–52^. The specific conclusions can be processed through two strategies. The first one is from specific (*e.g*., sparrow) to specific (*e.g*., crow), in which participants may draw an inference based on similarity or typicality. The other is from specific (*e.g*., sparrow) to general (*e.g*., bird), and then from general to specific (*e.g*., crow), in which participants may make a general conclusion first, and then a specific conclusion. For example, to make an inference from ‘sparrow has property X’ to ‘crow has property X’, participants may make a general conclusion about birds, that is, a sparrow is a type of bird, and has property X, so birds might have property X. Then, they could make a specific conclusion, that is, birds have property X, a crow is a type of bird, so, a crow should have property X. These two strategies are likely to account for the present findings.

Secondly, when the arguments were composed of the items from different hierarchical levels, there were also significant differences in the plausibility between conditions. Specifically, when the conclusion items were composed of the same subordinate items, the premises items composed of the basic level had stronger plausibility than those composed of the superordinate items. Similarly, when the conclusion items were composed of the same superordinate levels, the premise items composed by the basic level items had stronger plausibility than those composed of subordinate items. These results are also in accordance with the spreading activation model, and further suggest that the greater distance between the premise and the conclusion items is, the lower plausibility will be. However, no such a difference was found when the conclusion items were composed of the same basic items due to the fact that the distances from superordinate to basic condition and from subordinate to basic condition might be similar. This view was further supported by the stronger plausibility of arguments composed of adjacent hierarchical levels (*e.g*., subordinate-basic, superordinate-basic, and basic-subordinate) relative to those composed of more distant hierarchical levels (*e.g*., subordinate-superordinate, superordinate- subordinate).

In addition to the behavioral results, the modulations of N400 and P300 amplitudes as electrophysiological measurements also reflected that the distances between hierarchical levels significantly affect the process of the category-based induction. Specifically, the N400 amplitudes elicited by basic-subordinate condition were significantly more negative relative to the superordinate-subordinate condition. Furthermore, larger P300 amplitudes were elicited by superordinate-basic conditions relative to both the basic- basic condition, and the subordinate-basic condition. These results may be caused by different reasoning distances, which could be evaluated for validity and plausibility^53,54^. That is, when the hierarchical level of the premises (*e.g*., superordinate) was higher than that of the conclusions (*e.g*., basic), the conclusion must be correct. In contrast, when the hierarchical level of the premises (*e.g*., basic) was lower than that of the conclusions (*e.g*., superordinate), the conclusion was not necessarily correct. Therefore, for the two arguments composed of hierarchical levels with reverse directions the inductive strengths were different, which might affect the P300 amplitude^4,5^.

To further examine this point of view, we compared ERP amplitudes in conditions where the arguments were composed of different hierarchical levels with reverse directions. In sum, the results revealed that reasoning from higher hierarchical levels to lower hierarchical levels (*i.e*., basic-subordinate, superordinate-basic, and superordinate- subordinate conditions) elicited larger P300 amplitudes relative to that from lower hierarchical levels to higher hierarchical levels (*i.e*., subordinate-basic, basic- superordinate, and subordinate-superordinate conditions). Combined with the finding that P300 amplitudes were markedly larger in the superordinate-basic relative to subordinate-basic condition, all these results suggested that the modulation of P300 amplitudes might reflect the processing of different types of arguments (*e.g*., inductive and deductive reasoning)^7,55^, or arguments with different inductive strength^5^.

Such effects, however were not embodied in N400 amplitudes. In fact, we found that the N400 amplitudes elicited by the superordinate-subordinate condition were significantly smaller than those elicited by the basic-subordinate condition. It is likely that the level of generalisation and degree of familiarity in the superordinate (*e.g*., animal) category are higher than those in the basic (*e.g*., bird) category, the reasoning processing is more likely to be automatically activated from the superordinate to the subordinate category than that from the basic to the subordinate category. This finding was consistent with recent ERP studies, which found that the acquisition and processing of superordinate level concepts was even earlier than basic level concepts^39,56,57^. For example, Large *et al*.^39^ found that superordinate categorisations were performed more quickly than basic level categorisations, which elicited more positive amplitude at 320–420 ms relative to those at a basic level.

In addition, it is notable that considering that judgments about the plausibility were not equal to accuracy, we did not exclude the trials with implausible judgments when we analysed the ERP data. Thus, there were no significant correlations between the behavioural data (the proportion of “plausibility” responses and RT) and the N400 and P300 amplitudes. In fact, in our recent experiment, when only arguments reasoning from higher level categories to low level categories were included in the task, were the amplitudes of P300 indeed correlated with the proportion of “plausibility”. Based on previous studies and our results^7,36^, the P300 effects in present study might reflect the inductive strength of inductive reasoning. Meanwhile, it is worth noting that the P300 and N400 components emerged almost at the same time window, but with different regions. Because of the limitations of brain wave localization, in the current study, we cannot declare that the P300 and N400 components were absolutely different or the same one. But according to the current data analysis and statistic results (Fig. 2), as well as the results of the direction effect of reasoning only found on the amplitude of P300 (Fig. 3), we may infer that the two components represented different process of recognition, which also obtained in different regions of brain. However, this may worth to further investigate in the future.

## Conclusion

The present findings yield new insights into the processing of inductive reasoning by integrating the hierarchical categories with category-based property reasoning task. In the subordinate conclusion condition, the basic premise elicited a larger N400, versus the superordinate promise; in the basic conclusion condition, the superordinate promise elicited a larger P300 relative to both the basic premise and subordinate premise, indicating the modulation of hierarchical levels on the category-based induction. Furthermore, the P300 amplitude elicited by reasoning from a higher to a lower level was larger than those in the reverse directions, which is more likely to reflect the inductive strength or processing confidence. Overall, the diverging evidence from P300 and N400 effects suggested that the hierarchical levels of premises had critical regulatory roles in category-based induction.
